# Supplementary material for: Early crustal processes revealed by the ejection site of the oldest martian meteorite
Source: Nat Commun. 2022 Jul 12;13:3782. doi: 10.1038/s41467-022-31444-8 (PMC9276826; doi:10.1038/s41467-022-31444-8)
Supplement: Supplementary file 1 — Supplementary Information [file 41467_2022_31444_MOESM1_ESM.pdf]

Supplementary Information

EARLY CRUSTAL PROCESSES REVEALED BY THE EJECTION SITE OF THE OLDEST MARTIAN METEORITE

Lagain et al.

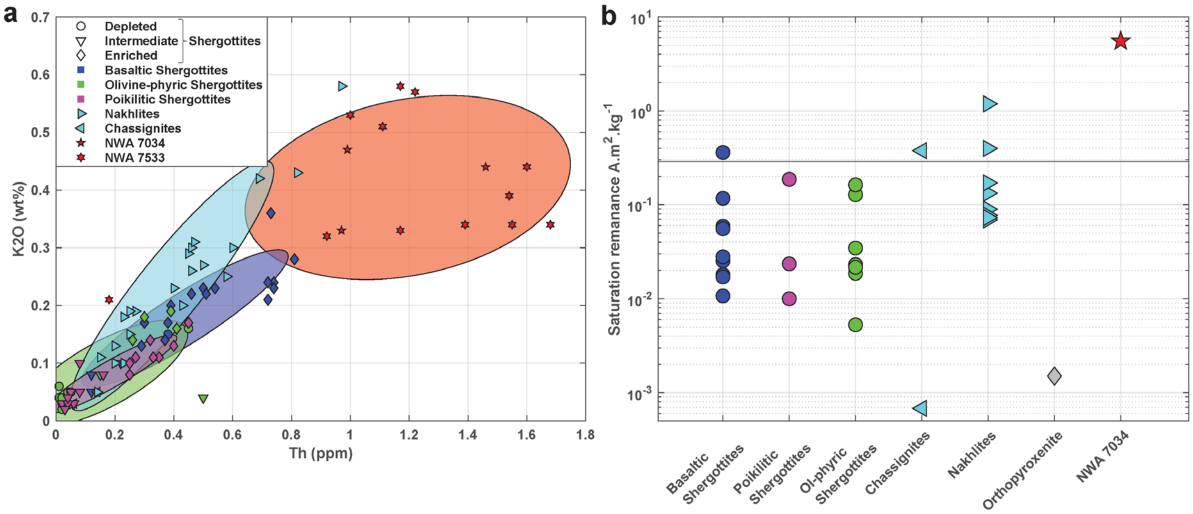

Supplementary Figure 1 | Elemental and magnetic characteristics of the regolith breccia. a: Concentration in K and Th in the regolith breccia compared to other groups of martian meteorites[2]. Ellipses correspond to one standard deviation of the data for each meteorite group (basaltic, olivine-phyric and poikilitic shergottites, nakhlites and chassignites, and the regolith breccia). **b**: Remanent magnetization of martian meteorites[7]. The horizontal line corresponds to the threshold value that is required to account for martian magnetic anomalies, i.e. 0.29 $A.m^2.kg^{-1}$ (ref. 7).

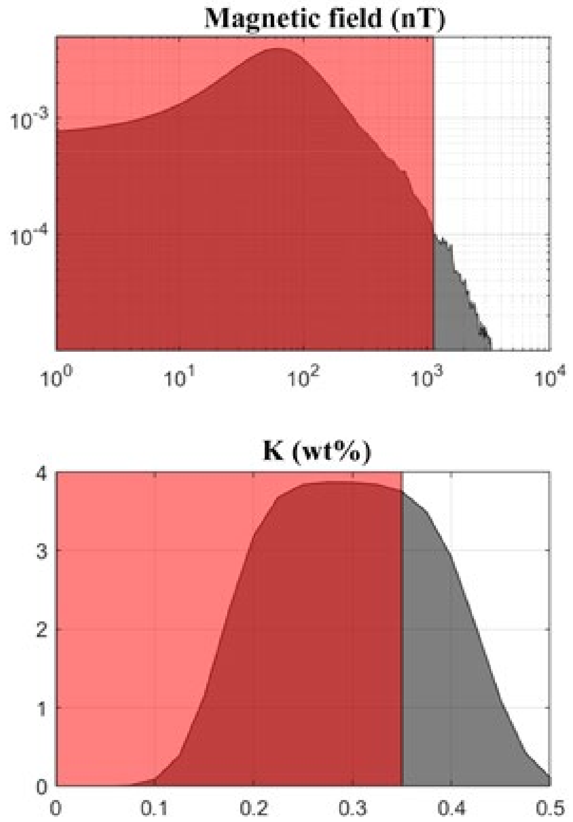

Supplementary Figure 2 | Probability density function from kernel density estimation of values of the four orbital datasets considered here[27-29]. Red areas correspond to the values below +1σ, considered here as low values: magnetic intensity = 1129.8 nT, equivalent magnetization = 2.00 $A.m^2.kg^{-1}$, K = 0.35 wt% and Th = 0.72 ppm. The distribution of the magnetic field and equivalent magnetization[27] are skewed positively with a large number of small values and includes a few major values, which result in the 1σ being greater than mode for those distributions.

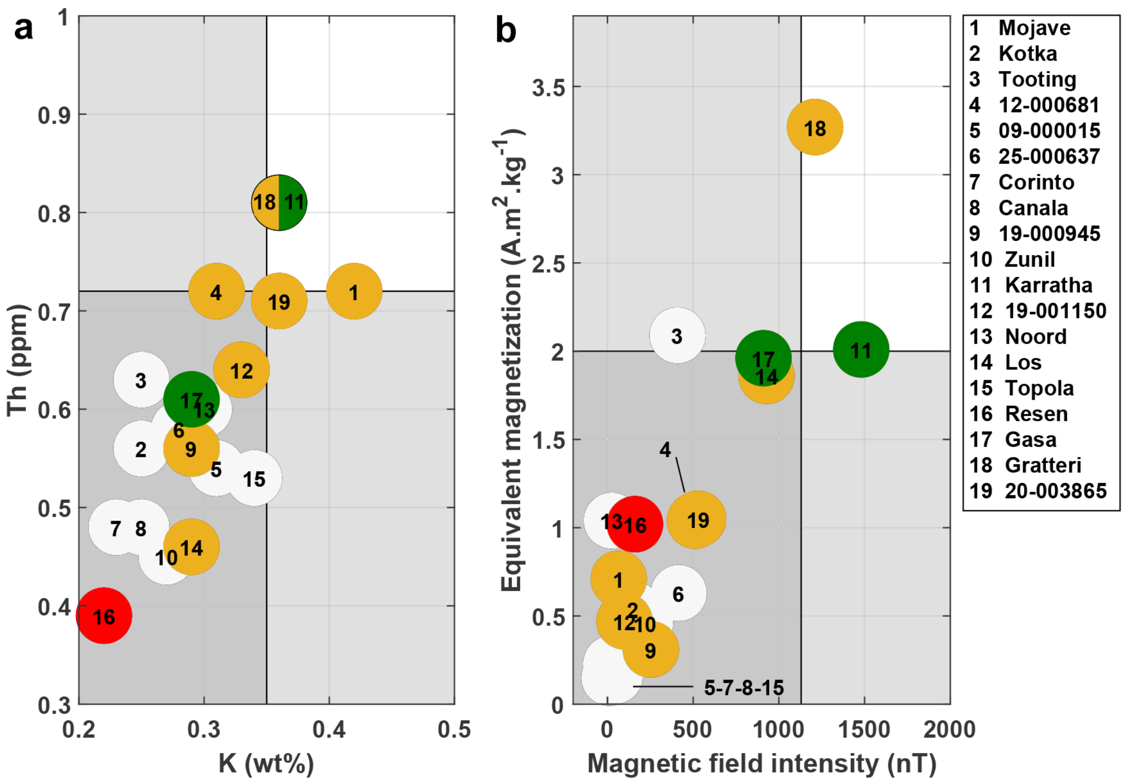

Supplementary Figure 3 | Abundance in K and Th (a) and equivalent magnetization and magnetic field intensity (b) of terrains surrounding each of the 19 young crater candidates. Differences between pixel values of each dataset associated with each crater and those obtained from the bilinear interpolation are negligible (Supplementary Tables 1 and 2). Only the pixel values are represented here (Supplementary Table 1). Grey areas correspond to one standard deviation of the respective dataset (in red in Supplementary Fig. 2), used as a threshold to distinguish low (grey areas) and high values (white areas). Each point is color-coded as a function of the superposition relationship criteria. In green, the crater respects the two criteria, i.e. the crater is located on a Noachian geological unit[30] and superposed on the material of an Amazonian impact crater (cavity or ejecta). In orange and red, only one of these two criteria is respected, orange craters are located on Noachian geological and red craters are superposed on the material of an Amazonian impact crater. In white, none of the superposition relationship criteria is respected. K and Th concentrations surrounding Karratha (label *11*) and Gratteri (label *18*) craters are similar. In comparison, K and Th concentration of NWA 7034 are respectively 0.3-0.6 wt% and 0.9-1.7 ppm (ref. 2) and its saturation remanence is 5.53 $A.m^2.kg^{-1}$ (ref. 7).

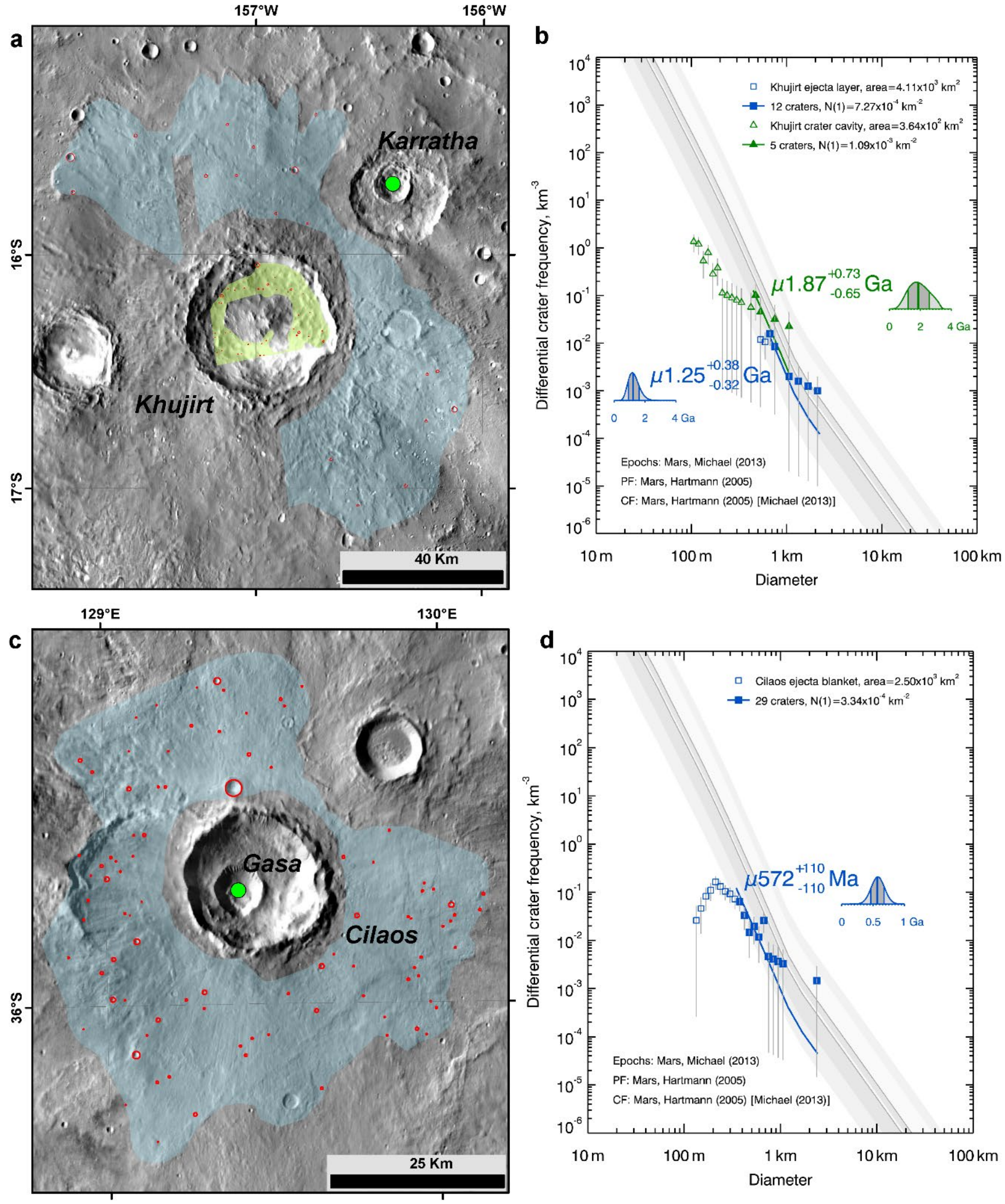

Supplementary Figure 4 | Crater counts and impact model ages of Khujirt and Cilaos craters, the host terrains of Karratha and Gasa crater respectively. a: Counting areas associated with the ejecta blanket and cavity of the 40 km crater (Khujirt) adjacent to Karratha. Red circles are impact craters superposed on the ejecta blanket of Khujirt crater (in blue) and on the crater cavity (in green). **b**: CSFD and model age derivation of the Khujirt impact event: the ejecta blanket (in blue, for D > 500 m), and the crater cavity (in green, for D > 100 m). **c**: Impact craters (red circles) superposed on the Cilaos ejecta blanket (in blue). **d**: CSFD and model age derivation of the Cilaos impact event (in blue, for D > 250 m). Background: THEMIS Day IR (Thermal Emission Imagery System, Day Infra-Red) mosaic (https://astrogeology.usgs.gov/search/map/Mars/Odyssey/THEMIS-IR-Mosaic-ASU/Mars_MO_THEMIS-IR-Day_mosaic_global_100m_v12).

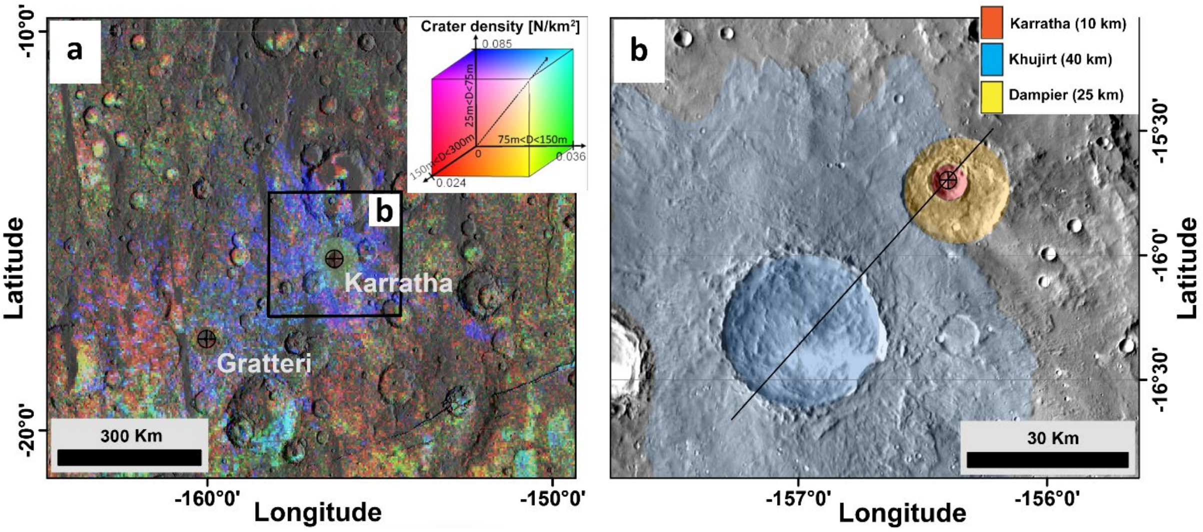

Supplementary Figure 5 | Regional crater density from ref. 13 and local geological context of the NWA 7034 launch site. a: Regional crater density map for D < 300 m identified by the CDA. Colors indicate crater densities of specific diameter ranges[18]. Blue rays around Karratha correspond to areas dominated by secondary craters <75 m in diameter[18]. **b**: Local geological context of Karratha crater (in red) and location of the cross section shown in Fig. 3a. Karratha (formed 5-10 Ma ago) is superposed on the ejecta blanket of Khujirt crater (formed ~1.5 Ga ago) shown in blue, that has partially filled the cavity of Dampier crater shown in yellow. Note that ejecta material from Khujirt crater within Dampier crater is not shown for clarity. Background: THEMIS Day IR (Thermal Emission Imagery System, Day Infra-Red) mosaic (https://astrogeology.usgs.gov/search/map/Mars/Odyssey/THEMIS-IR-Mosaic-ASU/Mars_MO_THEMIS-IR-Day_mosaic_global_100m_v12).

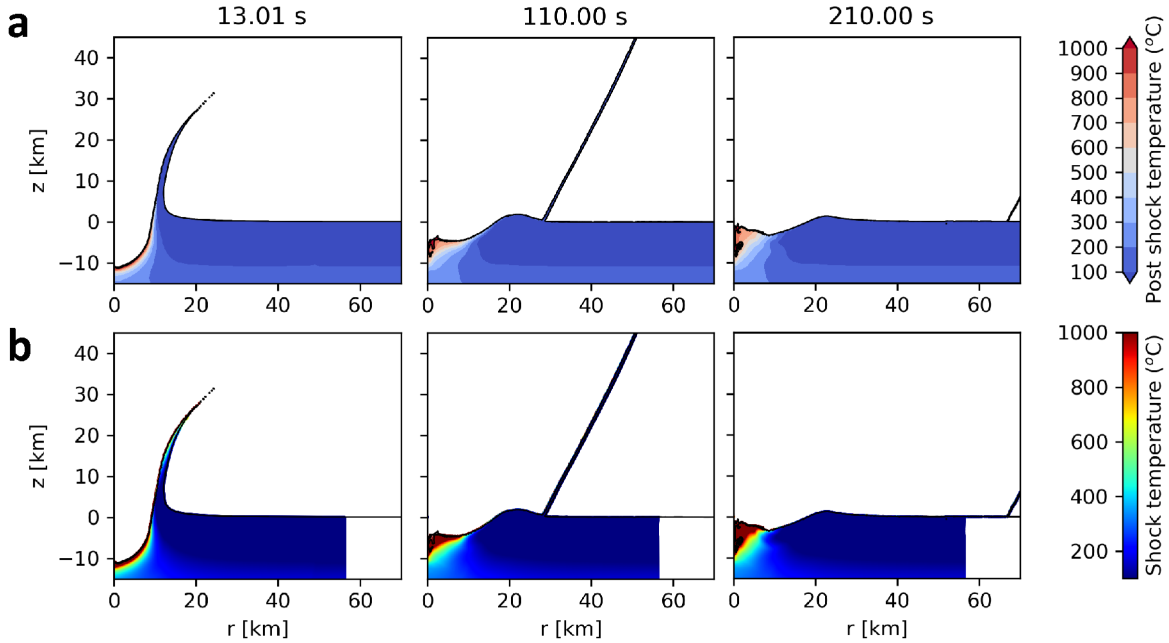

Supplementary Figure 6 | Illustration from iSALE 2D simulation of crater formation and rocks to be deposited following the Khujirt crater formation, near the Karratha site. A 4.5 km impactor striking Mars at 9.6 km/s has been used to model the formation of a 40 km impact crater. Output of the simulation at three different time steps (from left to right: 13, 110 and 210 seconds after the impact) are shown to illustrate a) post-shock temperatures and b) shock temperatures.

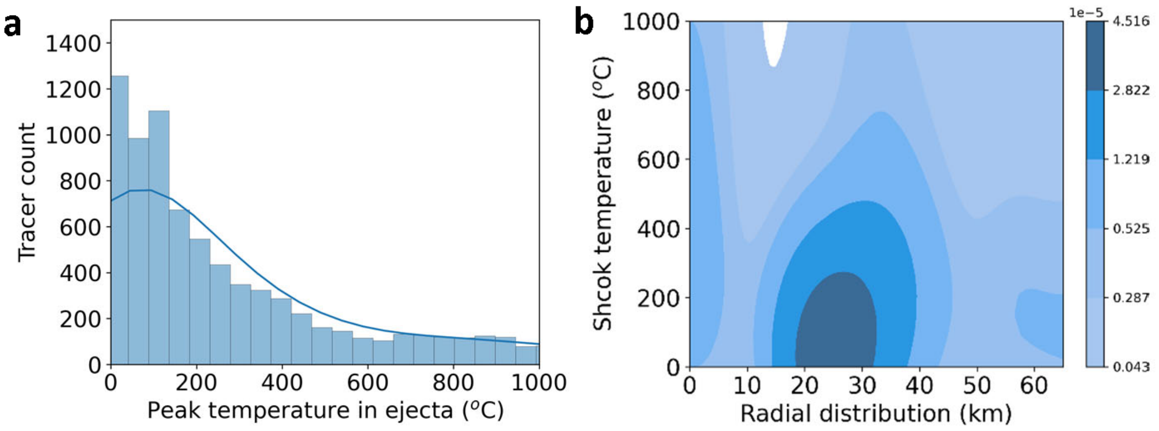

Supplementary Figure 7 | Temperature distribution within the ejecta of Khujirt crater from iSALE 2D simulation. a: Distribution of the shock temperature in proximal ejecta blanket estimated from the tracer particles that tracked the ejecta from launch to deposition, within 60 km distance from the crater center. It reaches up to >1000°C with higher temperatures being less frequent. **b**: 2D kernel density estimator plot of the distribution of ejecta compared with the likelihood of shock temperatures in the ejecta at these radial distances. Most ejecta is deposited around the rim, hence the highest density. The shock temperatures in the ejecta blanket range from 0 to >1000°C across the entire proximal ejecta distance as well as where Karratha crater formed, 55 km away from the Khujirt crater center.

Name	Long	Lat	D (km)	Noachian terrain	Unit	Superposed on Amazonian crater material	Magnetic intensity (nT)	Equivalent Magnetization (A.m^2.kg^{-1})	K (wt%)	Th (ppm)
Mojave (1)	7.5	-33.0	58	X	mNh		65.74	0.71	0.42	0.72
Kotka (2)	19.3	169.9	39		lAv		148.55	0.54	0.25	0.56
Tooting (3)	23.2	-152.2	29		lAv		408.93	2.09	0.25	0.63
12-000681 (4)	5.2	21.2	19	X	mNh		510.75	1.04	0.31	0.72
09-000015 (5)	19.3	-99.9	19		AHv		21.48	0.23	0.31	0.54
25-000637 (6)	-43.5	-101.5	17		eHv		416.09	0.63	0.28	0.58
Corinto (7)	16.9	141.7	14		AHv		10.82	0.15	0.23	0.48
Canala (8)	24.4	-80.1	11		eHh		35.73	0.2	0.25	0.48
19-000945 (9)	-19.7	-3.3	11	X	eNh		253.27	0.31	0.29	0.56
Zunil (10)	7.7	166.2	10		lAvf		219.82	0.46	0.27	0.45
Karratha (11)	-15.7	-156.4	10	X	mNh	X	1480.54	2.01	0.36	0.81
19-001150 (12)	-29.4	-8.7	9	X	eNh		99.05	0.47	0.33	0.64
Noord (13)	-19.3	-11.3	8	X	mNh		24.68	1.04	0.3	0.6
Los (14)	-35.1	-76.2	8	X	mNh		928.04	1.86	0.29	0.46
Topola (15)	15.8	-92.2	8		AHv		42.7	0.16	0.34	0.53
Resen (16)	-27.9	108.9	7		eHv	X	160.32	1.02	0.22	0.39
Gasa (17)	-35.7	129.4	7	X	eNh	X	910.64	1.96	0.29	0.61
Gratteri (18)	-17.7	-160.1	7	X	mNh		1211.29	3.27	0.36	0.81
20-003865 (19)	-11.0	25.9	3	X	mNh		529.3	1.05	0.36	0.71

Supplementary Table 1 | Summary of location, size and superposition relationship of the 19 crater candidates, and the magnetic intensity, magnetization, K and Th abundance of their host terrains. Only the pixel value associated with each dataset is reported here (Methods). For a graphical representation, readers are referred to the Supplementary Fig. 3. Numbers associated with each name correspond to the labels shown on Fig. 2a and Supplementary Fig. 3.

Name	Longitude	Latitude	D (km)	Magnetic intensity (nT)	Equivalent Magnetization ($A.m^2.kg^{-1}$)	K (wt%)	Th (ppm)
Mojave (1)	7.5	-33.0	58	201.18	1.03	0.39	0.69
Kotka (2)	19.3	169.9	39	146.08	0.54	0.23	0.52
Tooting (3)	23.2	-152.2	29	471.48	0.32	0.26	0.66
12-000681 (4)	5.2	21.2	19	547.78	0.71	0.33	0.7
09-000015 (5)	19.3	-99.9	19	21.42	0.23	0.3	0.54
25-000637 (6)	-43.5	-101.5	17	316.06	0.41	0.28	0.56
Corinto (7)	16.9	141.7	14	28.57	0.28	0.23	0.5
Canala (8)	24.4	-80.1	11	32.94	0.23	0.26	0.48
19-000945 (9)	-19.7	-3.3	11	237.99	0.31	0.32	0.59
Zunil (10)	7.7	166.2	10	184.45	0.7	0.25	0.46
Karratha (11)	-15.7	-156.4	10	1130.06	6.68	0.35	0.78
19-001150 (12)	-29.4	-8.7	9	181.51	0.62	0.32	0.66
Noord (13)	-19.3	-11.3	8	93.48	0.62	0.31	0.58
Los (14)	-35.1	-76.2	8	933.54	2.1	0.29	0.48
Topola (15)	15.8	-92.2	8	44.72	0.13	0.32	0.55
Resen (16)	-27.9	108.9	7	225.67	0.81	0.25	0.41
Gasa (17)	-35.7	129.4	7	787.69	1.8	0.3	0.62
Gratteri (18)	-17.7	-160.1	7	1444.89	3.27	0.37	0.85
20-003865 (19)	-11.0	25.9	3	515.54	1.05	0.36	0.71

Supplementary Table 2 | Bilinear interpolation values of the magnetic intensity, magnetization, K and Th abundance of the 19 crater candidate host terrains. Numbers associated with each name correspond to the labels shown on Fig. 2a and Supplementary Fig. 3.

Surface temperature (K)	210
Surface temperature gradient (K.m^{-1})	1.50x10^{-2}
Lithospheric thickness (m)	1.50x10^{5}
Gravity (m.s^{-2})	3.711
Object material	dunite
Object type	SPHEROID
Object velocity (m.s^{-1})	9.60x10^{3}
Target material	basalt
Thermal profile	CONDCONV
Compression rate	100

Supplementary Table 3 | Input iSALE 2D parameters used for modelling the 40 km impact crater shown in Supplementary Fig. 6.
